# Supplementary material for: A Scoping Review of Educational and Training Interventions on Parkinson’s Disease for Staff in Care Home Settings
Source: Nurs Rep. 2025 Jan 13;15(1):20. doi: 10.3390/nursrep15010020 (PMC11768021; doi:10.3390/nursrep15010020)
Supplement: Supplementary file 1 [file nursrep-15-00020-s001.zip › nursrep-3297139-supplementary.pdf]

Supplementary Material Table S1

Preferred Reporting Items for Systematic reviews and Meta-Analyses extension for Scoping Reviews (PRISMA-ScR) Checklist

SECTION	ITEM	PRISMA-ScR CHECKLIST ITEM	REPORTED ON PAGE #
**TITLE**			
Title	1	Identify the report as a scoping review.	1
**ABSTRACT**			
Structured summary	2	Provide a structured summary that includes (as applicable): background, objectives, eligibility criteria, sources of evidence, charting methods, results, and conclusions that relate to the review questions and objectives.	1
**INTRODUCTION**			
Rationale	3	Describe the rationale for the review in the context of what is already known. Explain why the review questions/objectives lend themselves to a scoping review approach.	1 & 2
Objectives	4	Provide an explicit statement of the questions and objectives being addressed with reference to their key elements (e.g., population or participants, concepts, and context) or other relevant key elements used to conceptualize the review questions and/or objectives.	2
**METHODS**			
Protocol and registration	5	Indicate whether a review protocol exists; state if and where it can be accessed (e.g., a Web address); and if available, provide registration information, including the registration number.	N/A
Eligibility criteria	6	Specify characteristics of the sources of evidence used as eligibility criteria (e.g., years considered, language, and publication status), and provide a rationale.	3
Information sources*	7	Describe all information sources in the search (e.g., databases with dates of coverage and contact with authors to identify additional sources), as well as the date the most recent search was executed.	3
Search	8	Present the full electronic search strategy for at least 1 database, including any limits used, such that it could be repeated.	Supplementary Material Table S2
Selection of sources of evidence†	9	State the process for selecting sources of evidence (i.e., screening and eligibility) included in the scoping review.	3 & 4
Data charting process‡	10	Describe the methods of charting data from the included sources of evidence (e.g., calibrated forms or forms that have been tested by the team before their use, and whether data charting was done independently or in duplicate) and any processes for obtaining and confirming data from investigators.	4
Data items	11	List and define all variables for which data were sought and any assumptions and simplifications made.	3

SECTION	ITEM	PRISMA-ScR CHECKLIST ITEM	REPORTED ON PAGE #
Critical appraisal of individual sources of evidence§	12	If done, provide a rationale for conducting a critical appraisal of included sources of evidence; describe the methods used and how this information was used in any data synthesis (if appropriate).	4
Synthesis of results	13	Describe the methods of handling and summarizing the data that were charted.	4
**RESULTS**			
Selection of sources of evidence	14	Give numbers of sources of evidence screened, assessed for eligibility, and included in the review, with reasons for exclusions at each stage, ideally using a flow diagram.	4 & 5
Characteristics of sources of evidence	15	For each source of evidence, present characteristics for which data were charted and provide the citations.	5 & 6
Critical appraisal within sources of evidence	16	If done, present data on critical appraisal of included sources of evidence (see item 12).	7
Results of individual sources of evidence	17	For each included source of evidence, present the relevant data that were charted that relate to the review questions and objectives.	7-10
Synthesis of results	18	Summarize and/or present the charting results as they relate to the review questions and objectives.	7-10
**DISCUSSION**			
Summary of evidence	19	Summarize the main results (including an overview of concepts, themes, and types of evidence available), link to the review questions and objectives, and consider the relevance to key groups.	10 & 11
Limitations	20	Discuss the limitations of the scoping review process.	12 &13
Conclusions	21	Provide a general interpretation of the results with respect to the review questions and objectives, as well as potential implications and/or next steps.	13
**FUNDING**			
Funding	22	Describe sources of funding for the included sources of evidence, as well as sources of funding for the scoping review. Describe the role of the funders of the scoping review.	13

JBI = Joanna Briggs Institute; PRISMA-ScR = Preferred Reporting Items for Systematic reviews and Meta-Analyses extension for Scoping Reviews.
* Where *sources of evidence* (see second footnote) are compiled from, such as bibliographic databases, social media platforms, and Web sites.
† A more inclusive/heterogeneous term used to account for the different types of evidence or data sources (e.g., quantitative and/or qualitative research, expert opinion, and policy documents) that may be eligible in a scoping review as opposed to only studies. This is not to be confused with *information sources* (see first footnote).
‡ The frameworks by Arksey and O'Malley (6) and Levac and colleagues (7) and the JBI guidance (4, 5) refer to the process of data extraction in a scoping review as data charting.
§ The process of systematically examining research evidence to assess its validity, results, and relevance before using it to inform a decision. This term is used for items 12 and 19 instead of "risk of bias" (which is more applicable to systematic reviews of interventions) to include and acknowledge the various sources of evidence that may be used in a scoping review (e.g., quantitative and/or qualitative research, expert opinion, and policy document).

From: Tricco AC, Lillie E, Zarin W, O'Brien KK, Colquhoun H, Levac D, et al. PRISMA Extension for Scoping Reviews (PRISMAScR): Checklist and Explanation. Ann Intern Med. 2018;169:467–473. doi: 10.7326/M18-0850.

Supplementary Material Table S2

EMBASE Search Strategy

Search ID	Search Terms	Number of Results
S40	S10 AND S23 AND S38	169
S39	S10 AND S23	1749
S38	S24 OR S25 OR S26 OR S27 OR S28 OR S29 OR S30 OR S31 OR S32 OR S33 OR S34 OR S35 OR S36 OR S37	961127
S37	Staff Development	2286
S36	Staff Education	4288
S35	Online Education	2715
S34	Educational Intervention	14110
S33	Continuing Professional Development	4229
S32	CPD	10223
S31	Staff Training	17141
S30	Learning	271626
S29	E-Learning	6964
S28	Self-Directed Learning	1656
S27	Training	129196
S26	Nursing Education	91816
S25	Educational Activities	5237
S24	Education	504015
S23	S11 OR S12 OR S13 OR S14 OR S15 OR S16 OR S17 OR S18 OR S19 OR S20 OR S21 OR S22	90224
S22	Residential Home Staff	7
S21	Nursing Home Staff	1237
S20	Care Home Staff	631
S19	Aged Care Facility	570
S18	Care Homes	4984
S17	Care Home	5007
S16	Long Term Care Facility	3613
S15	Long Term Care Facilities	7196
S14	Nursing Home	65123

S13	Nursing Homes	26133
S12	Residential Home	8144
S11	Residential Homes	1041
S10	S1 OR S2 OR S3 OR S4 OR S5 OR S6 OR S7 OR S8 OR S9	2783600
S9	Movement Disorder	31160
S8	Parkinson	222221
S7	Corticobasal Degeneration	3994
S6	Multiple System Atrophy	390
S5	Progressive Supranuclear Palsy	9415
S4	PD	2623564
S3	Parkinsonian	20510
S2	Parkinson’s Disease	204311
S1	Parkinsons	179754

Supplementary Material Table S3

Study Characteristics

Study	Country	Setting	Participants	Appraisal Score	Aim	Key Findings
Ashraf et al, 2012	England	Care homes	15 – care home staff	6/9	To use a Care Home education programme including a disease specific module on Parkinson's Disease to improve care home staff knowledge and practice in care homes	A structured education programme including general and disease-specific modules leads to improvement of staff knowledge and implementation of good practice in care homes.<br><br>Pre and post course knowledge tests showed an increase in test score post education programme<br><br>Post programme feedback gained via Likert style questionnaire, all participants stated learning would be shared with colleagues and eight participants gave examples of change in practice as a result of attending the course
Chenoweth et al, 2013	Australia	Acute/Sub-Acute Care Wards and Residential Aged Care Facilities	217 Acute/Sub-Acute Ward nurses and 127 Residential Aged Care Staff including Registered Nurses, Enrolled Nurses and specially trained nursing assistants	7/9	To determine the impact of a Parkinson's medicine education program on nurses' knowledge and practices in two settings where people with Parkinson's Disease are cared for, hospitals and residential aged care facilities	Nurses in residential aged care facilities had higher perceived and actual knowledge pre-test than hospital nurses and these levels increased at post program follow up. Both hospital and residential aged care facility staff were satisfied with the program as an education and support vehicle in management of Parkinson's Disease

Coles et al, 1995	England	Public and Private Residential Homes	150 care staff from 23 care homes	4/9	To evaluate the Communicate training program for staff working in residential homes with people living with dysphasia and other communication difficulties	Comprises of two training sessions – Training 1 exploring communication through role play and exchange of information between trainer and participants and Training 2 which uses video material to illustrate communication disability and helping strategies. Optional component available to be added on PD if felt to suit the profile of a particular home. All participants reported finding the training useful. It is stated that managers were interviewed pre and post training and that care staff reported they learned effective communication, to understand stroke, dementia, aphasia and PD, to be more patient, to relate to residents, to listen better and to cope. However, there are no statistics provided around pre-intervention knowledge or practices or on how many participants had the optional training on PD.
Eriksson et al, 2016	Sweden	Nursing Homes	5 enrolled nurses and 5 nursing home residents with communication difficulties	7/9	To evaluate the effect of a communication partner training programme directed to enrolled nurses in nursing homes working with people with communication disorders	Only 1 of the residents included had communication difficulties due to PD, remaining residents had communication difficulties of other aetiology.<br><br>Communication partners set goals to be achieved through the training. PD communication partner recognised they were dominant in communication and set goals to assist the person with PD in expression in conversation. The person with PD reported improvement in functional communication after the intervention, whereas the PD communication partner reported a deterioration in communication from baseline. However, the person with PD's general health deteriorated between intervention and follow up and the communication partner felt this may have been responsible for the deterioration. Study is limited by sample size being too small to determine if communication partner training can improve

						communication with people living with PD in care homes
Makoutonina and Iansek, 2010	Australia	Residential facilities	118 residential facility staff from 9 different facilities	6/9	PD education program to improve knowledge of residential facility staff, providing care for people living with PD, and determine if this translates into improvement in resident care, particularly in terms of falls	Curriculum focused on rehabilitation strategies, general PD knowledge, medication issues and accessing local specialist services. Staff knowledge improved after education from baseline and was maintained at 12 month follow up. Fall rate decreased by 9% per month over the year but most improvement was seen within the first four months post intervention.
Oates et al, 2016	England	Not specified	158 PD Nurse Specialists, 69 Parkinson's UK staff and 53 Care Home staff	6/9	To explore the models of training delivery and interaction between PD nurse specialists, Parkinson's UK and Care Home Staff when caring for a person living with Parkinson's in a care home.	64% of Parkinson's UK staff reported negative experiences for people living with PD of respite or care home placements which were frequently linked to a perceived lack of specialist training on PD for care home staff, with positive experiences reported being attributed to well trained staff. 96% of care home staff reported at some point having cared for a person living with PD. Falls, communication difficulties and physical difficulties were reported as the most difficult symptoms to manage. Only 59.62% of care home staff reported having had any form of training regarding caring for people living with PD. Care home staff who had not received training, average confidence level was 5.9/10 whereas care home staff who had had training had an average confidence level of 7.7/10. Most respondents felt more training could improve the care they provide for people living with PD. All groups of participants acknowledged that lack

						of training was a major barrier to providing quality care for people living with PD in care homes.
Wong and Luthra, 2019	United States of America	Long Term Care Facilities	Nurses in Long Term Care Facilities – number of participants not specified	6/9	To educate nurses in long term care facilities on non-motor symptoms of PD, to improve long term care facility nurses' recognition of non-motor symptoms and identify interventions for non-motor symptoms	Nurses included in study not aware of PD related non-motor symptoms prior to intervention. Post intervention survey results suggest an immediate knowledge increase on non-motor symptoms, however low response rate to post intervention survey limited evaluation of intervention
